# Хронический аутоиммунный тиреоидит — «сигнальное заболевание» в составе мультиорганного аутоиммунного синдрома

**DOI:** 10.14341/probl13361

**Published:** 2023-08-31

**Authors:** Е. А. Трошина

**Affiliations:** ФГБУ "Национальный медицинский исследовательский центр эндокринологии" Минздрава России

**Keywords:** аутоиммунные заболевания щитовидной железы, аутоиммунные полигландулярные синдромы, мультиорганный аутоиммунный синдром, хронический аутоиммунный тиреоидит, аутоиммунные заболевания

## Abstract

Текущее столетие объявлено ВОЗ «веком аутоиммунных заболеваний», которых к сегодняшнему дню насчитывается более ста. Естественное течение любого аутоиммунного заболевания характеризуется прогрессированием от латентной и субклинической стадий к клинической и связано с наличием специфических циркулирующих аутоантител. В течение жизни человека с одним верифицированным аутоиммунным заболеванием существует высокая вероятность последовательной манифестации других аутоиммунных патологий. Каждый четвертый пациент с хроническим аутоиммунным тиреоидитом заболевает аутоиммунными нетиреоидными патологиями, и наоборот, среди лиц с нетиреоидными аутоиммунными болезнями часто встречается хронический аутоиммунный тиреоидит. Современные представления о патогенетических механизмах развития и прогрессирования аутоиммунных заболеваний позволяют рассматривать хронический аутоиммунный тиреоидит в качестве «сигнальной патологии» при мультиорганном аутоиммунном синдроме.

## Abstract (en)

The current century has been declared by the WHO as the «century of autoimmune diseases», of which there are more than a hundred today. The natural course of any autoimmune disease is characterized by progression from a latent and subclinical to a clinical stage and is associated with the presence of specific circulating autoantibodies. During the life of a person with one verified autoimmune disease, there is a high probability of consecutive manifestation of other autoimmune pathologies. Every fourth patient with chronic autoimmune thyroiditis develops autoimmune non-thyroid pathologies during his life, and vice versa, chronic autoimmune thyroiditis is present among people with non-thyroid autoimmune diseases. Modern ideas about the pathogenetic mechanisms of the development and progression of autoimmune diseases make it possible to consider chronic autoimmune thyroiditis as a «signal pathology» in the multiorgan autoimmune syndrome.

Хронический аутоиммунный тиреоидит (ХАИТ) был впервые описан Хашимото в 1912 г. На сегодняшний день известно несколько вариантов течения этого заболевания. По морфологическим особенностям выделяют гипертрофическую форму АИТ (классический вариант ХАИТ, при котором доминирует лимфоидная инфильтрация) и атрофическую форму АИТ (доминируют признаки фиброза). Очевидно, что между известными вариантами заболевания существуют тонкие различия генетического и/или патогенетического характера, но имеется и много общего, вследствие чего их вполне обоснованно объединяют под одним названием аутоиммунного заболевания щитовидной железы (АЗЩЖ) — «хронический аутоиммунный тиреоидит».

ХАИТ является HLA-ассоциированным заболеванием, однако его атрофическая и гипертрофическая формы связаны с разными гаплотипами.

У родственников первой линии родства пациентов с ХАИТ риск развития этого же заболевания в 9 раз выше, чем в общей популяции. Ежегодная заболеваемость ХАИТ во всем мире составляет 0,3–1,5 случая на 1000 человек. Распространенность ХАИТ наиболее высока у женщин и достигает 2–11% в зависимости от возраста. Среди взрослых лиц в 90% случаев ХАИТ выявляется именно у женщин [1].

ХАИТ является, как и прочие аутоиммунные заболевания, результатом нарушения иммунорегуляции в сочетании с органической дисфункцией, которая, в свою очередь, представляет собой следствие антигенспецифической атаки, дополняемой недостаточной супрессией (и, следовательно, активацией) лимфоцитов, действие которых направлено на антигены на тиреоцитах (клетках-мишенях), а также в сочетании с выработкой различных цитокинов, воздействующих на клетки-мишени с близкого расстояния. Нарушение иммунологической аутотолерантности лежит в основе формирования ХАИТ [2].

Регуляторные Т-лимфоциты (Treg) играют ключевую роль в поддержании аутотолерантности и иммунного гомеостаза. Они оказывают супрессивное воздействие на остальные клетки иммунной системы, тем самым осуществляя контроль иммунного ответа и супрессию воспалительных реакций путем прямого подавления и модулирования функции эффекторных Т-клеток. При аутоиммунных процессах, в частности при ХАИТ, отмечаются снижение числа и функциональной активности Treg, а также несостоятельность фермента каспазы, участвующего в обеспечении их функционала [3]. Известно, что в патогенезе гипертрофической формы ХАИТ в большей степени задействованы Th1 (клеточный иммунитет), а в патогенезе его атрофической формы — Th2 (гуморальный иммунитет).

Хелперные T-лимфоциты подразделяются на подгруппы Th1 и Th2 в зависимости от способа выработки ими цитокинов: клетки Th1 выбрасывают интерферон гамма (IFN-γ), фактор некроза опухоли альфа и интерлейкин-2 (ИЛ-2) и создают воспалительный эффект, тогда как клетки Th2 «помогают» B-лимфоцитам, в первую очередь вырабатывая интерлейкины (ИЛ-4, -5 и -6). Также установлено, что три подгруппы стромальных клеток щитовидной железы, эндотелиальные клетки ACKR1+, миофибробласты CCL21+ и фибробласты CCL21+, вносят вклад в микроокружение ткани щитовидной железы при ХАИТ. Предположительно они могут способствовать переносу лимфоцитов из крови в ткани щитовидной железы, а фибробласты Т-клеточной зоны CCL21+ также могут способствовать формированию третичных лимфоидных органов, характерных для ХАИТ, заболевания, при котором показано также наличие воспалительных макрофагов и дендритных клеток, экспрессирующих высокие уровни ИЛ-1β в щитовидной железе. Это, в свою очередь, может способствовать разрушению тиреоцитов при ХАИТ [4]. Кроме того, увеличение количества дендритных клеток (ДК) в инфильтратах щитовидной железы регистрируется при всех основных разновидностях АЗЩЖ, причем как зрелых, локализованных в соединительной ткани, так и незрелых ДК, группирующихся вокруг тиреоидных фолликулов. У пациентов с ХАИТ показатели концентрации субпопуляций ДК в периферической крови значительно ниже, чем у здоровых людей, а сами ДК характеризуются повышенной выработкой интерферона-альфа в условиях in vitro под воздействием стимуляции, и, в отличие от периферических плазмоцитоидных ДК, ДК щитовидной железы демонстрируют более высокую экспрессию Fas-лиганда.

Подчеркнем еще раз, — Тh1 активируют макрофаги и цитотоксические Т-лимфоциты, которые опосредованно, через цитотоксические механизмы, разрушают фолликулярные клетки щитовидной железы. Механизм активации запускается через действие провоспалительных цитокинов, которые индуцируют экспрессию целого ряда молекул, вызывающих активацию апоптоза фолликулярных клеток щитовидной железы. Также отмечено, что при ХАИТ в ткани щитовидной железы увеличивается экспрессия Th1-цитокинов — IFN-γ и ИЛ-2, что также коррелирует с локальными воспалительными реакциями. В свою очередь, Th2 играют большую роль в дифференцировке и пролиферации В-клеток. При избыточности действия Th2-цитокинов возможно образование из В-клеток плазматических клеток, которые вырабатывают антитела к антигенам щитовидной железы [5].

Важно отметить, что лимфоциты, несущие рецепторы, способные распознавать аутоантигены, образуются постоянно, и в норме эти клетки должны быть элиминированы или инактивированы, как только они распознают антигены, чтобы предупредить их патогенное действие. Однако некоторые антигены скрыты от иммунной системы, т.к. ткани органов, в которых они локализуются, не сообщаются с кровью или лимфой и являются априори «забарьерными». Таким образом, эти аутоантигены не индуцируют толерантность, но и не вызывают иммунного ответа и по существу «игнорируются» иммунной системой. К таким органам частично относится щитовидная железа, и в норме невозможно или крайне сложно индуцировать иммунный ответ, вводя в такие иммунологически привилегированные органы антиген. Однако если антигены высвобождаются по каким-то причинам из самих этих тканей, например вследствие травмы, или инфекции, или за счет генетически обусловленной «поломки» в функционале Т- и В- регуляторных клеток, то возможен иммунный ответ, приводящий к длительному воспалению тканей и их повреждению. Именно это и лежит в основе развития ХАИТ.

В свою очередь, B-лимфоциты секретируют антитела, в том числе аутоантитела. Продукция антител способствует поддержанию оптимального уровня CD4(+) Т-клеточной активации, так как В-клетки выступают в качестве антиген-представляющих клеток (АПК) и оказывают другие иммуномодулирующие функции в иммунном ответе. Однако определенные В-клетки могут также подавлять иммунный ответ посредством производства регуляторных цитокинов и непосредственно взаимодействуя с Т-клетками через клеточные контакты. Такой тип В-клеток называется регуляторными В-клетками (Breg), которые являются иммуносупрессивными и поддерживают иммунологическую толерантность. Снижение количества Breg или их несостоятельность ведет к срыву иммунологической толерантности, что также характерно для ХАИТ [5].

Почему же все-таки инициируется аутоиммунная агрессия? Ведь аутоиммунитет можно рассматривать в качестве физиологической реакции нормальной иммунной системы на аутоантиген, выработанный в результате воспалительного ответа на вирусную инфекцию, или на какой-то другой антиген, экспрессируемый в ткани-мишени, либо на повреждение тиреоцитов иной природы. Вирусный или микробный антиген, обладающий сходством с аутоантигеном (молекулярная мимикрия), способен запускать выработку аутоантител, которые вступают в перекрестную реакцию с аутоантигеном, после чего иммунный ответ вступает в реакцию с соответствующими структурами аутологичных клеток. Многими исследователями высказываются предположения, что ХАИТ «вероятно, провоцируется каким-то внешним фактором», например инфекцией, и этот фактор запускает экспрессию тиреоцитами HLA-DR, которая и приводит к развитию заболевания [6]. Однако все очевиднее становится тот факт, что ХАИТ представляет собой расстройство иммунорегуляции, обусловленное частичным нарушением антигенспецифической иммуносупрессии в сочетании с неспецифическими факторами окружающей среды, которые опосредованно оказывают воздействие на иммунную систему. Старт аутоиммунной атаки у генетически предрасположенного человека зависит от факторов окружающей среды, доступности антигенов посредством АПК и их характера, антигенпроцессирующих и антигенпрезентирующих генов, выработки различных цитокинов и их воздействия на клетки-мишени и иммунную систему, a также от роли различных иммунореактивных молекул, вырабатываемых иммунными клетками и клетками-мишенями после начала собственно иммунной атаки [7].

В качестве вероятных механизмов, лежащих в основе развития ХАИТ, рассматриваются:

Изолированное или содружественное влияние данных факторов ведет к повреждению тиреоцитов и запуску аутоиммунной агрессии [9].

Наиболее значимые аутоантигены щитовидной железы, вовлеченные в процесс развития ХАИТ, представлены ниже:

Следует отметить, что пероксидаза щитовидной железы является ведущим патогенетически значимым аутоантигеном. При ХАИТ в сыворотке крови больных выявляются в 90–100% случаев аутоантитела к ТПО. Наличие аутоантител к ТПО — показатель агрессии иммунной системы по отношению к собственному организму. В отличие от других, аутоантитела к ТПО являются комплементфиксирующими и непосредственно участвуют в комплементзависимой цитотоксичности, вызывающей усиление органоспецифической аутоагрессии. Высокая частота встречаемости аутоантител к ТПО наблюдается нередко при нетиреоидных заболеваниях: сахарном диабете (25%), пернициозной анемии (52%) и первичном билиарном циррозе (29%) [10].

Это указывает на полиорганный характер аутоиммунных заболеваний и демонстрирует значимость аутоантител к ТПО при различных аутоиммунных заболеваниях, а сам патогенез ХАИТ может стать некой «иллюстрацией-клише» для понимания подобных процессов в других органах при синдроме мультиорганных аутоиммунных поражений. Как уже было отмечено выше, провоцирующие факторы окружающей среды (стресс, инфекция, травма, лекарственные препараты, курение, старение) могут подавлять функционирование неспецифических Ts, усугубляя этим антиген-специфическую дисфункцию Ts, а формирующийся очаг аутоиммунного повреждения ткани ЩЖ (или любого другого органа) с течением времени рекрутирует в пораженную ткань все большую популяцию клонов неспецифических T-лимфоцитов [11].

ХАИТ является «вершиной айсберга» мультиорганного аутоиммунного синдрома (МАС) в том случае, когда имеет место генерализованная аномалия в функционировании T-клеток, приводящая к множественным клиническим расстройствам иммунорегуляции. Если же есть нарушение лишь в функционале антигенспецифических супрессорных T-лимфоцитов, ХАИТ (как и любое другое аутоиммунное заболевание) будет монопатологией. Тем не менее важно, что и неспецифические, и антигенспецифические супрессорные Т-лимфоциты могут обнаруживать совместное (аддитивное) действие, биологическая и клиническая суть этого явления — предмет дальнейших исследований, которые, вероятно прольют свет на патогенез МАС. Уже доказано наличие органоспецифического нарушения в функционировании супрессорных T-лимфоцитов при ХАИТ, независимо от нарушения функции ЩЖ [12][13].

Подобные процессы могут иметь место и при прочих аутоиммунных заболеваниях в их латентной фазе течения. Показано, что нарушения в антигенспецифических супрессорных Т-лимфоцитах (носящие частичный или относительный характер) вполне могут быть обусловлены дефектом презентации специфического антигена (возможно, по причине отклонений в антигенпрезентирующем гене, следствием которых является снижение активации специфических супрессорных клеток).

Имеются данные о сенсибилизации T-лимфоцитов при ХАИТ к интактным клеткам щитовидной железы либо к TГ, TПO или рТТГ [14]. Актуальные исследования фокусируются на определении того, какой именно эпитоп данных антигенных молекул является специальным сайтом связывания и активным сайтом для тиреоспецифических Т-клеток, а также для тиреоидных аутоантител.

Система HLA играет ключевую роль в процессировании и презентации антигенов. Презентация антигена генами HLA классов I и II является принципиально важным фактором развития аутоиммунных заболеваний, в т.ч. ХАИТ. При этом запускаются сложное взаимодействие между антигеном, присутствующим на клетках-мишенях и вырабатываемым этими клетками, АПК, хелперно-индукторными T-лимфоцитами CD4, эффекторными T-лимфоцитами и супрессорными (регуляторными)/цитотоксическими T-лимфоцитами CD8, и выработка активированными таким образом T-лимфоцитами интерферона-гамма (ИФН-γ), a также определенных цитокиновых профилей (т.e. клеток Th1 и Th2) и связующих молекул (например, молекул межклеточной адгезии (ММА-1), белков теплового шока и т.д.). Все они также играют важную роль в координации иммунного ответа. Например, экспрессия ММА-1 и белка клеточной адгезии 1-го типа тиреоидными клетками, которая в норме не осуществляется, но при ХАИТ носит очевидный характер и усиливает активацию лимфоцитов. Тиреоцитный белок теплового шока-72 также экспрессируется только клетками, пораженными аутоиммунной атакой, но не в норме. Подобная экспрессия может оказывать некоторый иммуномодулирующий эффект в форме усиления связывания иммуноглобулина. Тиреоидные клетки могут вследствие цитокиновой стимуляции или комплементной атаки продуцировать и некоторые другие иммуноактивные молекулы (например, простагландин E2, ИЛ-6 и ИЛ-8), что дополнительно усиливает тиреоцитно-иммуноцитную сигнализацию [15][16].

Любопытно, что сами тиреоциты экспрессируют HLA-DR. Бесспорно, что экспрессия HLA-DR тиреоцитами является результатом «кооперации» местных АПК и инфильтрирующих сенсибилизированных и активированных T-лимфоцитов CD4 в выработке ИФН-γ, который, в свою очередь, стимулирует клетки щитовидной железы. Данные факты согласуются с тем, что клетки щитовидной железы изначально находятся в норме и экспрессируют такие молекулы, как HLA-DR, ММА-I лишь вследствие иммунного расстройства, хотя и могут производить эффект усиления иммунного ответа [2][17].

Антигены гистосовместимости ответственны не только за предрасположенность к развитию заболеваний, но и за сроки возникновения, характер течения и исход патологического процесса. Иммуногенетические факторы имеют важное значение в развитии ХАИТ. Его ассоциации отмечены с HLA-B8, -DR3, -DR4.

И все-таки, является ли наличие значимой внутренней аномалии в тиреоците или в аутоантигенах щитовидной железы обязательным условием для стимулирования развития ХАИТ? Казалось бы, да, поскольку аутоиммунитет — это реакция иммунной системы на аутоантиген, выработанный в результате повреждения ткани-мишени или на какой-то другой антиген, экспрессируемый в ней. Однако у здоровых людей лимфоциты обладают необходимыми генами и способностью вырабатывать антитиреоидные антитела в ответ на усиленный выброс антигена щитовидной железы из воспаленных и поврежденных тиреоцитов, но сам по себе подобный избыточный выброс антигена в иммунную систему к АИЗЩЖ не приведет. Реакция иммунной системы в этом случае должна быть и будет в норме преходящей (что и происходит при вирусном поражении ткани щитовидной железы при подостром тиреоидите или коронавирусной инфекции, когда наблюдается преходящий адекватный иммунный ответ на высвобождение антигена, но развития ХАИТ не происходит у подавляющего количества пациентов [7]. Только у очень ограниченного круга лиц (вероятно, имеющих генетическую предрасположенность к развитию аутоиммунных патологий) вирусный или микробный антиген, обладающий сходством с аутоантигеном (молекулярная мимикрия), способен запускать выработку аутоантител, которые вступают в перекрестную реакцию с аутоантигеном, после чего иммунный ответ вступает в реакцию с соответствующими структурами любых аутологичных клеток, в т.ч. тиреоцитов. Факты наличия врожденных генетически обусловленных аномалий в тиреоците у людей не подтверждены, многие исследователи склонны полагать, что клетки пораженной аутоиммунным процессом щитовидной железы человека в сущности, нормальны и не имеют признаков исходных генетических нарушений. Следовательно, возникновение АЗЩЖ зависит в первую очередь от наличия исходных (генетических) нарушений в иммунной системе. Это положение подтверждается частым наличием сочетанных аутоиммунных патологий у одного и того же человека. Причем сочетание именно ХАИТ с другими аутоиммунными патологиями позволяет рассматривать именно это заболевание щитовидной железы в качестве «сигнального», а его выявление должно насторожить клинициста в отношении большой вероятности наличия или возникновения все новых и новых аутоиммунных заболеваний у одного и того же пациента [18].

Известно, что поражение двух и более эндокринных желез дает основание для диагноза «аутоиммунный полигландулярный синдром» (АПС). Однако, помимо поражения органов эндокринной системы, в состав АПС может входить и аутоиммунное поражение неэндокринных органов. В этой связи в последние годы предлагается новая классификация АПС, в которой все чаще выделяется вариант, именуемый мультиорганным аутоиммунным синдромом (АПС/МАС-3) [19].

В целом АПС представлены большим спектром аутоиммунных заболеваний и подразделяются на два основных типа: АПС 1 типа — орфанное моногенное заболевание с манифестацией в детском возрасте, связанное с дефектом аутоиммунного гена-регулятора (AIRE), расположенного в длинном плече 21-й хромосомы (развивается в детском возрасте, характеризуется первичной хронической надпочечниковой недостаточностью (1-ХНН), гипопаратиреозом, кандидозом кожи и слизистых оболочек); АПС взрослых, ассоциированный с HLA-системой и CTLA-4 (подразделяется на 2 и 3 типы). АПС 2 типа характеризуется обязательным наличием надпочечниковой недостаточности и АЗЩЖ, а также сахарного диабета 1 типа. АПС 3 типа включает обязательное АЗЩЖ, комплекс прочих аутоиммунных заболеваний эндокринных желез (исключая надпочечниковую недостаточность) и неэндокринных органов. Ранее выделяли также АПС 4 типа (1-ХНН в сочетании с одной или несколькими второстепенными (как правило, неэндокринными) аутоиммунными патологиями, исключая основные составляющие АПС 1 и 2 типов). В настоящее время АПС 4 типа вошел в состав АПС 3, который предложено переименовать в АПС/МАС-3 (аутоиммунный полигландулярный синдром/множественный аутоиммунный синдром-3). При присоединении новых компонентов синдрома один тип АПС взрослых может быть переклассифицирован в другой [20][21].

В итоге, в зависимости от комбинаций аутоиммунных поражений, выделяют четыре подтипа АПС/МАС-3, а именно:

Если распространенность АПС 1 и 2 типов в популяции известна и составляет около 1,4–4,5 на 100 000 населения, то данные о распространенности АПС/МАС-3 отсутствуют. Однако, по мнению разных авторов и с учетом латентных форм заболевания, распространенность АПС/МАС-3 может доходить до 150 на 100 000 населения. Как правило, манифестация заболевания происходит в возрасте 20–60 лет и наиболее часто приходится на 3–4-ю декаду жизни [20][22].

Принципиальным моментом в диагностике АПС 2 и АПС/МАС-3 является раннее выявление их компонентов на его латентной стадии, которая может быть представлена субклиническим и потенциальным вариантами (в первом случае имеет место наличие одного аутоиммунного заболевания в сочетании с одним и более серологическими маркерами других основных составляющих синдрома плюс субклиническое нарушение функции второго органа-мишени; во втором случае диагностируется наличие одного эндокринного аутоиммунного заболевания в сочетании с антителами к другим органам, но без нарушения их функции).

Итак, сегодня очевидно, что АПС — это не только патология, характеризующаяся преимущественным поражением эндокринных желез. Для синдрома характерны мультиорганные аутоиммуные заболевания, как эндокринные, так и неэндокринные (желудочно-кишечные, кожные, неврологические или ревматологические, пульмонологические и другие), при этом ХАИТ — самая частая находка при всех вариантах АПС 2 и 3 типов. Этот факт неоднократно подтвержден многочисленными исследованиями. Так, при скрининговых обследованиях практически здорового населения у 10–13% женщин и 2–3% мужчин обнаруживаются аутоантитела к щитовидной железе. Более того, сывороточные концентрации антител к ТПО и ТГ коррелируют и с наличием очагового тиреоидита, обнаруженного в образцах ткани щитовидной железы, полученных при аутопсии у лиц, которые ранее не имели нарушения ее функции [21][23].

Примерно 1/3 женщин и 1/4 мужчин с клиническими, субклиническими или потенциальными аутоиммунными заболеваниями предрасположены к развитию других аутоиммунных патологий, а АПС/МАС-3 является наиболее частым АПС во всем мире [24]. Важно отметить, что генетическая предрасположенность к АПС/МАС-3 может наблюдаться более чем у одного члена семьи, различные аллели HLA класса II коррелируют с АПС/МАС-3 в зависимости от ассоциированных с ним аутоиммунных заболеваний. Другими вовлеченными генами являются те, которые кодируют лимфоидную тирозин-фосфатазу и цитотоксический антиген-4, ассоциированный с Т-лимфоцитами [25].

Как было упомянуто ранее, наличие ХАИТ или носительство антител к аутоантигенам щитовидной железы может являться сигнальным состоянием, позволяющим заподозрить у пациента АПС/МАС-3, и поводом для проведения детального обследования и (или) системного динамического наблюдения. Не исключено, что наблюдение в динамике за лицами — носителями антитиреоидных антител (в т.ч. даже антител к тиреоглобулину) является оправданным. Нужно отметить, что хотя антитела к тиреоглобулину (АТ-ТГ) и обладают меньшей чувствительностью и специфичностью для постановки диагноза любого АЗЩЖ, чем антитела к ТПО, именно они отражают начальный иммунный ответ и, следовательно, могут являться единственными антителами, присутствующими на самых начальных стадиях аутоиммунного процесса в щитовидной железе [26]. В свою очередь, старт аутоиммунного процесса в ткани ЩЖ может быть наиболее ранним маркером латентного АПС/МАС-3. При этом, несмотря на то, что аутоантитела ко многим органоспецифическим антигенам чаще выявляются у пациентов с АЗЩЖ, чем у здоровых людей, это не является отражением антигенной перекрестной реактивности, а скорее, может свидетельствовать о наследовании одного и более генов предрасположенности к патологиям — компонентам АПС/МАС-3, расположенным в непосредственной близости друг к другу на 6-й хромосоме. Совместное возникновение компонентов АПС/МАС-3 может быть объяснено и изменениями целого ряда сигнальных путей клеточно-щелочного баланса, что, в свою очередь, влияет на митохондриальный биогенез. Показано, например, что новые гены кислотно-основного механизма могут быть задействованы в патогенезе моногенных/полигенных аутоиммунных патологий и даже у пациентов без заболеваний желудочно-кишечного тракта. Определена совокупность носителей растворенных веществ, совместно экспрессируемых в тканях различных органов, пораженных аутоиммунными процессами, иллюстрирующая уникальное генетическое происхождение сочетанных эндокринных и неэндокринных патологий при АПС/МАС-3. Таким образом, формируется принципиально новый ландшафт причин развития аутоиммунных заболеваний [27].

Пациентам с ХАИТ рекомендуется проводить периодическое тестирование на другие аутоантитела в зависимости от их клинического статуса и/или семейного анамнеза [28].

В этом отношении весьма перспективным является метод мультиплексного анализа различных антител при подозрении как на АПС 1, так и на АПС взрослых, включая АПС/МАС-3. Такой метод обнаружения специфичных для АПС 1 аутоантител к интерферону омега, интерферону альфа-2-а, ИЛ-22 и органо-специфичных аутоантител, характерных для АПС взрослых (к 21-гидроксилазе, глутаматдекарбоксилазе, цитоплазматическому антигену островковых клеток, тирозинфосфатазе, ТГ, ТПО), был разработан и запатентован специалистами ГНЦ ФГБУ «НМИЦ эндокринологии» Минздрава России в партнерстве с НИИ молекулярной биологии РАН им. Энгельгардта [29][30]. В настоящее время панели органоспецифических антител для этого метода существенно расширены с учетом возможных неэндокринных компонентов АПС/МАС-3. После соответствующей регистрации использования разработанной диагностической методики для медицинских целей и ее валидизации она станет доступна в клинической практике, значительно сократив и удешевив обследование, в том числе и пациентов с подозрением на АПС/МАС-3. Особой когортой для применения созданного метода являются так называемые «ядерные семьи», члены которых имеют сочетанные аутоиммунные поражения эндокринных желез и неэндокринных органов, в связи с чем требуется постоянный контроль наличия/отсутствия маркеров, характеризующих развитие новых компонентов АПС/МАС-3. Осведомленность и раннее распознавание множественных ассоциаций аутоиммунных заболеваний, несомненно, должны быть в фокусе внимания клиницистов.

Принципиально важное значение имеют дальнейшие исследования, направленные на изучение гетерогенности АПС/МАС-3 и генетических механизмов, ответственных за развитие его подтипов. В свою очередь, результаты таких исследований, выполненных на больших выборках пациентов, позволят не только персонифицировать лечение, но и существенно улучшить раннюю диагностику синдрома, в т.ч. на самых ранних стадиях, и сформировать современные алгоритмы динамического наблюдения.

Таким образом, актуальность изучения фундаментальных закономерностей развития и прогрессирования аутоиммунных мультиорганных патологий обусловлена острой потребностью в создании многокомпонентной системы прогнозирования данных расстройств в ядерных семьях и популяции в целом, их предотвращения раннего выявления, и эффективной коррекции. Все это требует использования комплексного междисциплинарного подхода, включающего применение достижений иммунологии, молекулярной генетики, клеточной биологии и целого ряда других фундаментальных и клинических дисциплин. Принимая во внимание современную концепцию «сигнального заболевания» в составе мультиорганного аутоиммунного синдрома, в реальной клинической практике следует рассматривать наличие АИЗЩЖ, в первую очередь ХАИТ, в качестве вероятного повода для расширенного диагностического поиска прочих патологических состояний – компонентов синдрома.

## ДОПОЛНИТЕЛЬНАЯ ИНФОРМАЦИЯ

Источники финансирования. Статья подготовлена в рамках проекта, реализуемого по гранту РНФ «Научное обоснование, разработка и внедрение новых технологий диагностики коморбидных йододефицитных и аутоиммунных заболеваний щитовидной железы, в том числе с использованием возможностей искусственного интеллекта», No 22-15-00135, https://rscf/ru/project/22-15-00135/

Конфликт интересов. Автор декларируют отсутствие явных и потенциальных конфликтов интересов, связанных с публикацией настоящей статьи.

Участие автора. Автор одобрил финальную версию статьи перед публикацией, выразил согласие нести ответственность за все аспекты работы, подразумевающую надлежащее изучение и решение вопросов, связанных с точностью или добросовестностью любой части работы.
